# Characterization of circulating myeloma tumor cells by next generation flowcytometry in scleromyxedema patient: a case report

**DOI:** 10.1097/MD.0000000000020726

**Published:** 2020-07-02

**Authors:** Ruba Y. Taha, Saba Hasan, Firyal Ibrahim, Yannick Chantran, Hesham El Sabah, Siveen Sivaraman, Issam Al Bozom, Ahmad Al Sabbagh, Laurent Garderet, Halima El Omri

**Affiliations:** a Hamad Medical Corporation, Hematology and Medical Oncology Department; b Hamad Medical Corporations, department and Laboratory Medicine and Pathology, Doha, Qatar; c Department of Immunology, Hospital St Antoine, Paris, France; d Hamad Medical Corporations, Interim Translational research Institute iTRI, Doha, Qatar; e Maladies du sang CHU de l’AP-HP Hôpital St-Antoine 184 rue du fg St-Antoine 75571 Paris, France.

**Keywords:** biomarkers, immune response, immunoblotting, immunophenotyping, minimal residual disease, next generation flowcytometry, scleromyxedema

## Abstract

**Introduction:**

Scleromyxedema (rare cutaneous mucinosis), is characterized by the formation of lichenoid papules and presence of Serum monoclonal IgG in most cases, or all; after repeated testing.

**Patient concerns:**

: The patient is a 51-year-old male presented with thick, disfiguring elephant-like erythematous skin folds over the forehead, papular shiny eruptions over ears and trunk and waxy erythematous papules over arms and hands without dysphagia or respiratory or neurologic symptoms

**Diagnosis:**

: Skin biopsy from right arm was consistent with scleromyxedema. Serum cryoglobulin was reported negative. Complete blood count and routine blood biochemistry were normal. Thyroid function tests were normal. Serum protein electrophoresis and immunofixation showed monoclonal band of 14.5 g/L typed as IgG lambda.

**Interventions:**

: Our patient was refractory to lenalidomide however improved clinically on immunoglobulins infusions on monthly basis without change in the MGUS level.

**Outcomes:**

: NGF analysis revealed approximately 0.25% Lambda monotypic plasma cells in the bone marrow expressing CD38, CD138, and CD27 with aberrant expression of CD56 and were negative for CD45, CD19, CD117, and CD81. We also detected 0.002% circulating plasma cells (PCs) in peripheral blood.

**Conclusion:**

: The immunophenotype of circulating tumor cells (CTCs) remain close to the malignant PCs phenotype in the BM. Hence, we report NGF approach as a novel diagnostic tool for highly sensitive MRD detection in plasma cell dyscrasias including scleromyxedema.

## 1 Introduction

Treatment outcomes of multiple myeloma (MM) have progressed so much that have led to the implementation of new response criteria, including minimal residual disease (MRD) status as one of the most essential clinical endpoints.^[1]^ Currently, the landscape for multiple myeloma treatment have modified substantially, leading to increased complete response (CR) rates and survival.^[2–8]^ Still, most CR patients ultimately show relapse. Therefore, highly sensitive methods are needed for detection of minimal residual disease (MRD). Conventional 4–8-color flow cytometry (FCM), is the technique of choice for monitoring MRD in bone marrow (BM) of MM after therapy.^[9–15]^ Multiparameter flow cytometrys immunophenotyping is an anchor for monitoring of most hematologic malignancies. It has high relevance in differential diagnostic workup because of its steady and conclusive readout of plasma cell (PC) clonality and delivering of prognostic information in monoclonal gammopathies. The main function of FCM is measurement of intrinsic optical properties of particles, such as size or cytoplasmic complexity of a single blood or bone marrow cells, also the presence of intracytoplasmic or membrane protein within such a cell, by previous binding with a fluorochrome-coupled specific antibody.

**Figure 1:**
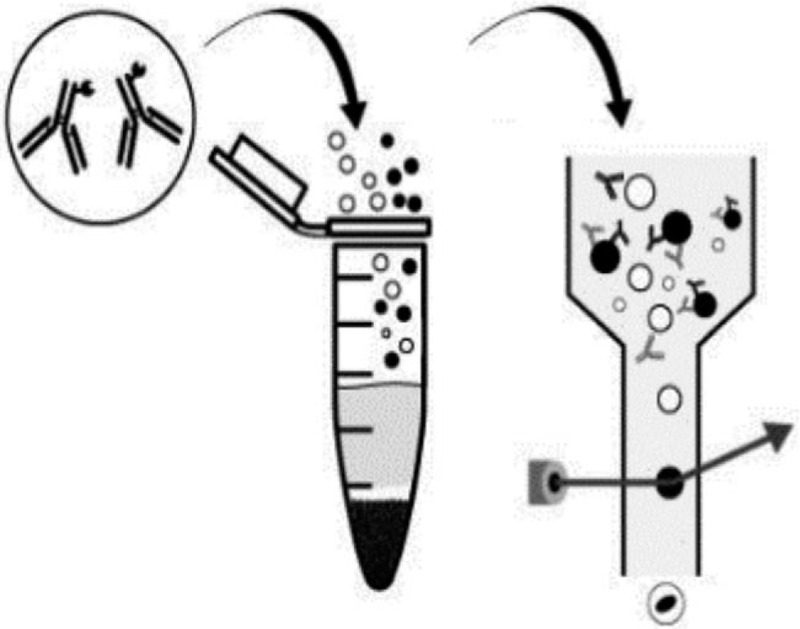
Fluorescence-activated cell sorting (FACS)s basic principle (Fluorochrome conjugated antibodies can be analyzed by Flowcytometry).

FCM is being a routine qualitative and quantitative technique, commonly used in standard clinical testing as well as in different scientific areas. In general, FCM is based on the analysis of light scattering characteristics of a cell suspension (size and granularity). The additional specific characteristics of the biological sample are obtained via the fluorescent probes used in the experiment^[16]^ (Fig. 1). The use of different fluorophores allows experimenters and researchers to analyze multiple parameters in a single assay. In hematology, FCM is a sensitive technique crucial for medical diagnosis and disease management. It allows different applications like DNA content analysis, immunophenotyping, and assessment of structural and functional properties of biological samples. Multi-color flow cytometry for MRD measurements in multiple myeloma can be considered applicable in all MM patients (95%) as compared to allele-specific oligonucleotide quantitative polymerase chain reaction (ASOqPCR) and next generation sequencing (NGS) (50%–90% cases).^[17]^ The superiority of applicability in flow-MRD is mainly due to the high level of primer annealing variability and the unpredictable amplification/quantitation results in NGS. On the other hand, PCR and NGS have higher sensitivity (10^−5^–10^−6^) compared to conventional flow-MRD (10^−4^). More recently, the limit of detection (LOD) was improved in next generation flow-MRD (10^−4^ and 10^−5^), making it as sensitive as PCR-based MRD methods at the condition of enough cell number should be measured.^[18]^ Next generation flow cytometry (NGF) is recently considered as a robust sensitive tool to evaluate monoclonal gammopathy of undetermined significance (MGUS) and MM.^[19]^

Scleromyxedema (SM) is a chronic, progressive, and potentially fatal mucinosis of the skin, displaying increased collagen and fibroblast proliferation with irregular distribution, along with involvement of various internal organs.^[20]^ Pathogenesis of this fatal mucinosis remains unclear. The high prevalence of monoclonal gammopathy (MGUS) in up to 80% of patients is an indication of a possible B-cell immune response to antigenic mucin deposits in the dermis.^[21]^ It was described by Rongioletti and Rebora (2001),^[21]^ as a generalized papular and sclerodermoid eruption, associated with monoclonal gammopathy (mostly IgG-λ paraproteinemia) and a triad of histological features: presence of mucin deposition within the upper and mid reticular dermis, fibroblast proliferation and fibrosis with the absence of a thyroid disorder.^[22,23]^ Histopathological analysis highlights a number of mucin deposits in papules and sclerotic malformations, comprising of thickened collagen fibers, due to which, lichenoid papules are formed which cause thickening and hardening of the tissue.^[24]^ Diagnosis depends on 4 criteria:

1. Papular cutaneous eruption in a scleroderma-like distribution;
2. Skin biopsy showing triad of dermal mucin deposition, proliferation of fibroblasts, and fibrosis;
3. Monoclonal gammopathy;
4. Absence of thyroid dysfunction.

The etiology of SM remains unknown. However, the pathogenesis of the disease has been suspected to be linked to the monoclonal. FCM is part of the initial diagnostic work-up, after assessment of patients clinical history and results of morphological assessment of blood and bone marrow, mainly because of its capacity to provide conclusive results within a specified period.

In the current study, we described the phenotypic characterization of clonal plasma cells in a patient with SM where the BM and the circulating plasma cells are examined. Additionally, the purpose of this article is to provide an overview of the methodology of NGF and to highlight some applications of this technique in clinical practice, specifically, as a tool for Scleromyxedema. The patient has provided informed consent for publication of the case.

## 2 Methods

The study was conducted with the approval of Hamad Medical Corporation (HMC) Institutional Review board (IRB) with a signed consent from the patient. Ethical approval was obtained from HMC-IRB (Study No: 14-226/14; JIRB No: 14-00089; QNRF No: NPRP7-916-3-237) for the research proposal entitled: Standardization of phenotypic and molecular techniques for characterization of circulating tumor cells and minimal residual diseases cells: understanding disease dissemination and chemoresistance, and approved on December 20, 2016.

Immunoblotting experiments were performed as follows: serum protein electrophoresis was performed in agarose gel (0.8%, pH 8.6) using a Sebia HYDRASIS, according to the manufacturer recommendations. After the migration and before the drying phase, the gel was taken out of the device and proteins were blotted on a nitrocellulose membrane during 10 minutes under a weight of 20 g/cm^2^. The membrane was then saturated during 1 hour with nonfat dry milk 5% H_2_O. After 3 washing steps with PBS-Tween 0.05%, membrane strips were incubated 1 hour with primary monoclonal antibodies (HP6053, HP6054, NL16, GOM2, ZG4, RJ4, TM15, HP6018, G7C, X3A8, HP6017. See Table 1 for specificity) diluted in PBS BSA 2%. After 3 washing steps with PBS-Tween 0.05%, membrane strips were the incubated 1 hour with secondary polyclonal anti-mouse IgG antibody coupled to the Alkaline Phosphatase (ALP), diluted in PBS BSA 2%. After washing steps, the presence of ALP was revealed by NitroBlue Tetrazolium / 5-bromo-4-chloro-3’-indolyphosphate system.

**Table 1:**
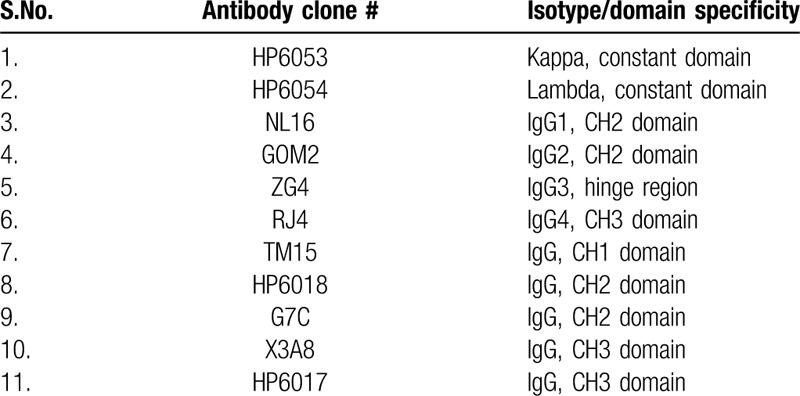
Monoclonal antibodies used for immunoblotting.

FCM was performed by next generation multiparametric flow cytometry utilizing BD Fortessa and analyzing the data using Infinicyt software (Cytognos SL). Both peripheral blood and bone marrow were evaluated by Bulk lyse-Stain-Wash protocol using a direct 8-color immunofluorescence combination; CD38 FITC, CD56 PE, CD45 PerCP-cy5.5, CD19 PE-Cy7, CD117 APC, CD81 APC-Cy7, CD138 Pacific Blue, CD27 BV510 for characterization of the immunophenotypic protein expression profile and a combination of CD38 FITC, CD56 PE, CD45 PerCP- Cyanine 5.5, CD19 PE-Cyanine 7, Kappa APC, Lambda APC-Cy7, CD138 - BV421, CD27 BV510 for characterization of cell clonality.

## 3 Results

We applied the NGF technique in the clinical setting of a case of scleromyxedema having MGUS as part of his presentation.

### 3.1 Case description

**Figure 2:**
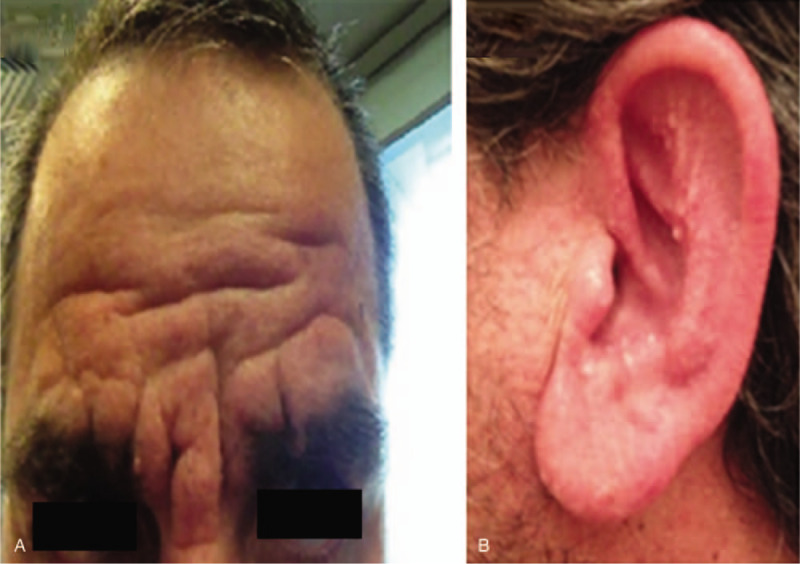
Patient disfiguring elephant like erythematous skin. (A) Erythematous thick skin folds over forehead. (B) Waxy and firm erythematous papules over ear.

**Figure 3:**
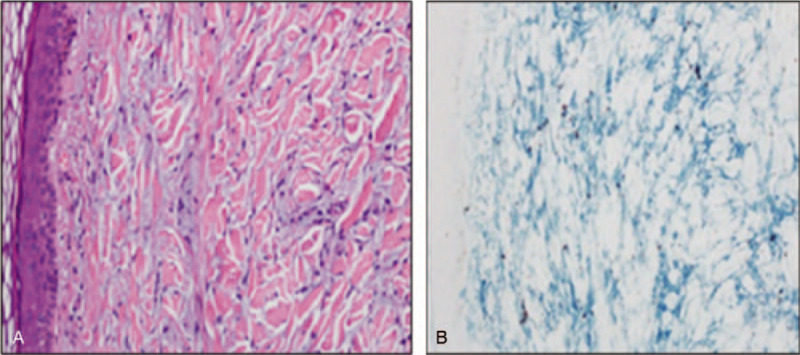
Skin Punch biopsy (A) The deposition of mucinous material (appears blue in between the pink collagen bundle in the reticular dermis, H&E stain(×200). (B) The mucinous material is highlighted by deep blue special staining colloidal iron stain (×200).

We describe a 51-year-old male with history of thick, disfiguring elephant like erythematous skin folds over the forehead, papular shiny eruptions over ears and trunk and waxy erythematous papules over arms and hands. No systemic manifestation, dysphagia or respiratory or neurologic symptoms were reported. On admission, sclerodermoid lesions, pseudosclerodermatous thickening of the exposed skin and thickening on the trunk and extremities were seen in the patient (Fig. 2A and 2B). Skin biopsy showed deposition of mucinous material compatible with scleomyxedema (Fig. 3A and 3B).

The laboratory investigations (Table 2) revealed mild normocytic normochromic anemia; hemoglobin 11.8 g/dl with normal white cell counts and platelet counts. Blood morphology showed increased rouleaux formation. Kidney, liver, thyroid functions were normal, calcium level was normal. Serum protein electrophoresis (SPEP) and immunofixation (IF) showed monoclonal band 14.5 gm/l typed as IgG lambda. Urine Bence Jones protein was negative for monoclonal protein. Beta2 microglobulin and lactate dehydrogenase were normal. Serum cryogolgulines were negative.

**Table 2:**
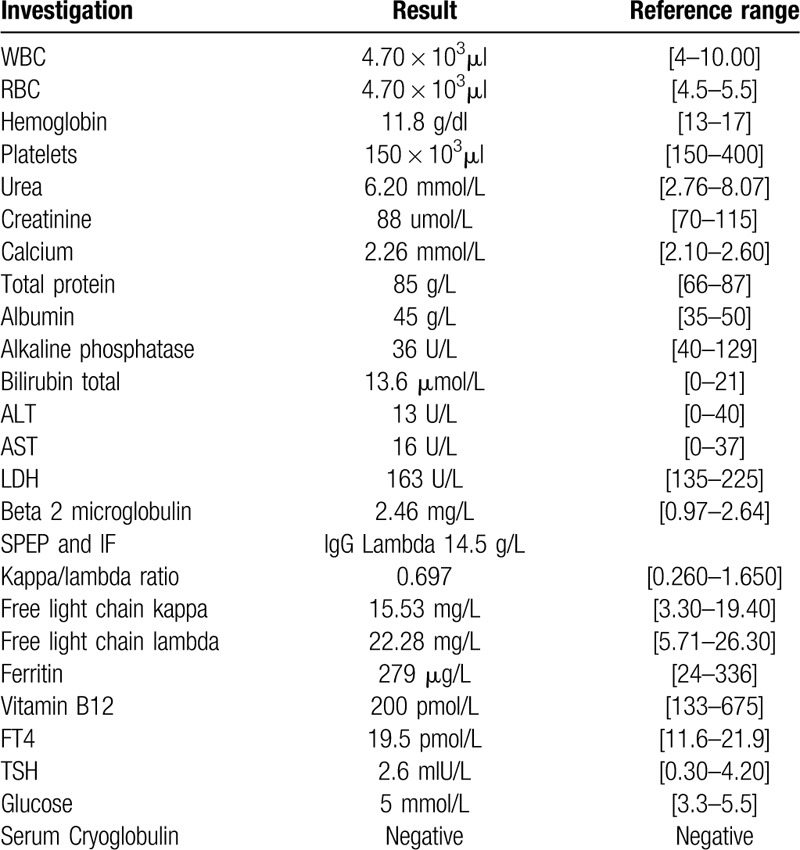
Results of patients diagnostic tests.

Radiologic investigations included ultrasound abdomen showed normal findings. Total body magnetic resonance imaging (MRI) did not reveal any focal osseous lesion. Echocardiography revealed normal parameters. All investigations excluded myeloma and could be explained by MGUS in the setting of scleromyxedema

### 3.2 Immunoblot study rational

**Figure 4:**
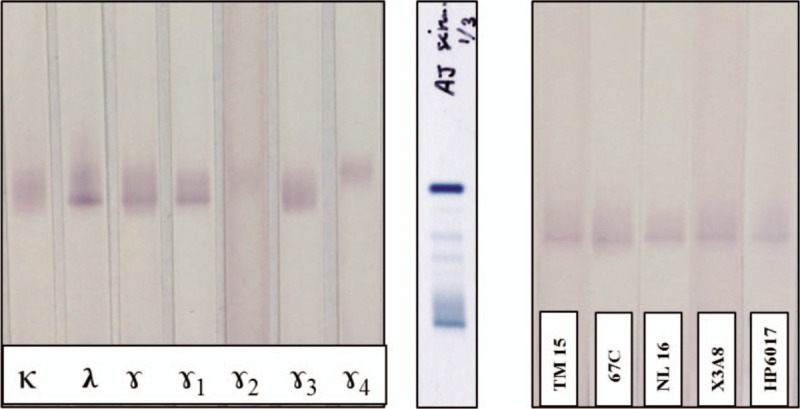
Gel electrophoresis showing identification of Immunoglobulin sub classes (A): first experiment was dedicated to the identification of Ig subclasses and light chain type. (B): second experiment was performed with antibodies specific for the 3 constant domains of the heavy chain.

A monoclonal immunoglobulin present in SM has been described decades ago by Kitamura et al (1979)^[25]^ as an IgG lambda with deletion of the heavy chain constant domain CH1. We performed immunoblotting experiments in order to investigate potential CH deletion in the patients monoclonal immunoglobulin. The first immunoblotting experiment with anti-IgG subclasses anti-sera showed a reactive band at the same migration as the monoclonal band on total protein electrophoresis with anti-Lambda, anti-IgG, and anti-IgG1 antibodies, but not with anti-Kappa, anti-IgG2, -IgG3, or -IgG4. We conclude that the monoclonal immunoglobulin belongs to the IgG1 subclass (Fig. 4A). The second immunoblotting experiment showed consistent immunoreactivity of the monoclonal band with monoclonal antibodies directed against IgG CH1, CH2, and CH3 domains (Fig. 4B). We exclude the complete deletion of a CH domain within this monoclonal immunoglobulin and conclude that partial deletion of a CH domain is unlikely.

**Figure 5:**
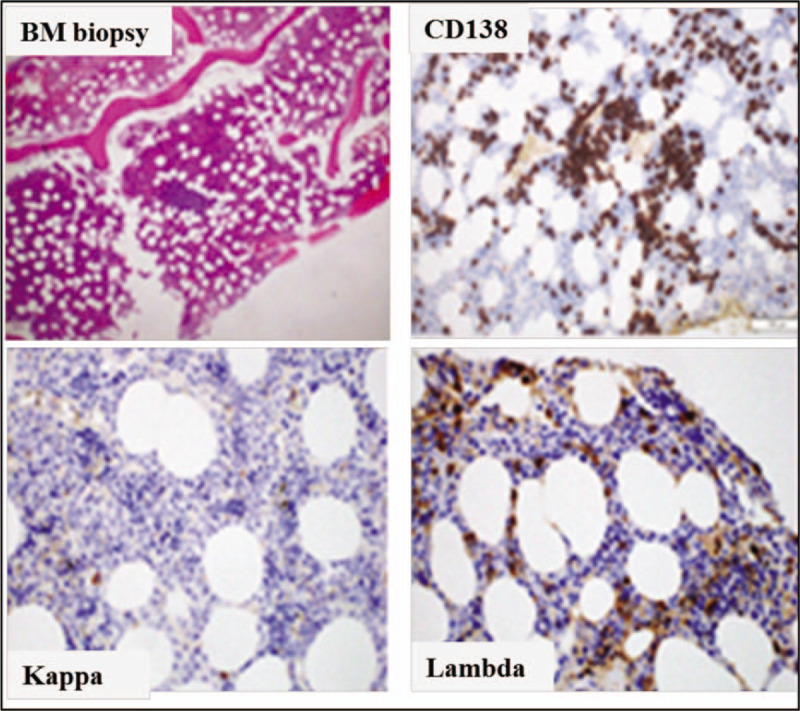
Bone marrow biopsy (H&E) 40×: cellular core biopsy. Immunohistochemistry on core biopsy (100×): CD138 immunostaining: positive, increased plasma cells scattered and in clusters. Kappa immunostaining: Rare positive cells, Lambda immunostaining: Many positive cells.

Bone marrow aspirate smear revealed active trilineage haemopoiesis with approximately 4% plasma cells, mostly mature, with occasional plasmablastic and binucleated forms. Bone marrow biopsy was cellular with adequate haemopoietic cells. Immunohistochemistry with CD138, Kappa and Lmabda revealed increased plasma cell comprising approximately 6% to 9% of bone marrow cellularity as, scattered, clusters, and aggregates, with Lambda restricted (Fig. 5).

**Figure 6:**
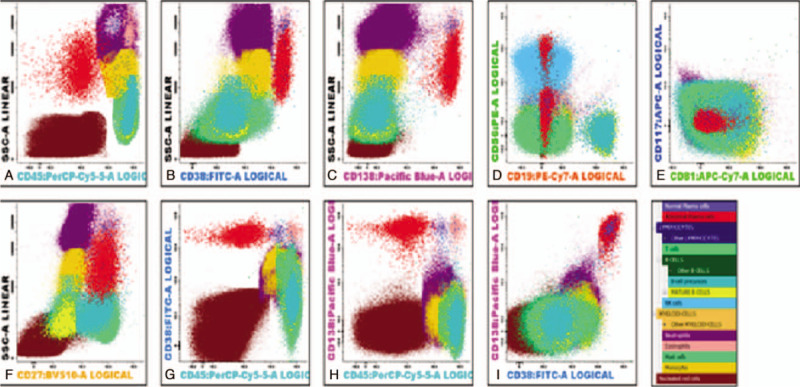
Bone Marrow next generation Flow cytometer: 0.25% plasma cells; Clonality: Lambda; Immunophenotype: A: CD45-, B: CD38++, C: CD138++, D: CD19-, E: CD56 Heterogeneous, F: CD27+, G: CD117-, H: CD81-.

**Figure 7:**
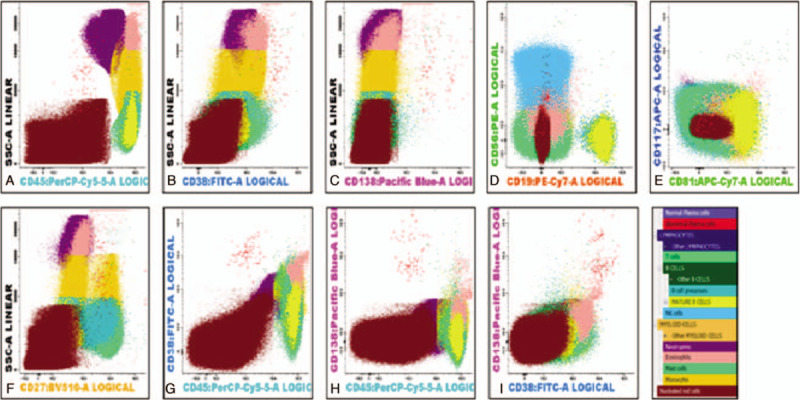
Peripheral Blood next generation Flow cytometer: 0.002% plasma cells; Clonality: Lambda. Immunophenotype: A: CD45-, B: CD38++, C: CD138 Heterogeneous, D: CD19-, E: CD56; Heterogeneous, F: CD27+, G: CD117-, H: CD81-.

Immunophenotyping analysis by NGF revealed approximately 0.25% Lambda monotypic plasma cells in the bone marrow expressing CD38, CD138, CD27 with aberrant expression of CD56 and were negative for CD45, CD19, CD117, and CD81. In the peripheral blood 0.002% circulating Lambda monotypic plasma cells were detected with similar immunophenotype expression (Figs. 6 and 7).

As evident from studies of Juan Flores et al (2016),^[26]^ myeloma PCs display phenotypes that deviate from those typically seen in normal PCs, including normal BM PCs. Markers that have been associated with informative aberrant antigen expression profiles for MRD monitoring in MM include: CD19, CD56, CD45, CD38, CD27, and to a less extent also CD20, CD28, CD33, CD117, and SmIg. On the other hand, the combination of these markers (or a subset of them) with cytoplasmic immunoglobulin (CyIg) lambda (L) and kappa (K) light chain staining may also contribute to establish the clonal nature of a population of suspicious PCs. Currently, additional markers are identified as aberrantly expressed by MM PCs in variable percentages of patients. Among these latter markers, CD81, CD200, CD54, and CD307 have emerged as most informative ones.

The patient was treated with lenalidomide 25 mg daily; The M band responded. However, skin lesions were refractory and progressing, the patient discontinued the drug after 1 year because of repeated infections and lack of efficacy. Subsequently the patient started second line treatment with IV immunoglobulin 2 g/kg divided over 3 days, he achieved favorable cutaneous response in-spite of persistent paraproteinemia.

## 4 Discussion

Scleromyxedema is a rare chronic disorder that may affect middle-aged adults (30 to 80 years old) at equal prevalence for both genders without any ethnic predominance.^[27,28]^ The etiology of the disease is still unclear. However, there are 4 diagnostic criteria commonly reported in SM patients

1. cutaneous mucinosis involving flesh-colored waxy rash with firm papules and sclerodermoid often in a linear array
2. microscopic triad (deposition of mucin, fibroblast proliferation)
3. monoclonal gammopathy, and absence of thyroid disease.^[28–30]^ Moreover, the disease has been reported to be associated with neurologic symptoms.^[31]^

Histologically, both scleromyxedema and lichen myxoedematosus show fibroblast proliferation and mucin (acid mucopolysaccharides) deposition in the superficial dermis between the collagen bundles with marked thickening of the dermis in case of scleromyxedema. In order to differentiate between these entities, thyroid dysfunction must be excluded and a monoclonal gammopathy shown in peripheral blood.^[32]^ The underlying mechanism and causes of scleromyxedema remain a challenge. However, the description about diagnostic criteria and treatment plan has increased the awareness and early recognition of the disease in recent years.^[33]^ Hence, there is no clarity in explanation of mucin deposition origin, paraprotein role and factors primarily implicated in the disease progression.^[34]^ Although, few cases of scleromyxedema were reported to be associated with neoplasms^[35]^ and bone marrow malignancies such as multiple myeloma,^[36]^ lymphoma,^[37]^ and myelomonocytic leukemia.^[38]^

MGUS is present in about 3% to 4% of normal individual above the age of 50 years of age.^[39]^ the risk of progression to MM or related disorders is around 1% per year ^[40]^. The presence of circulating tumor plasma cells in PB assessed by NGF at diagnosis emerges as a hallmark of disseminated plasma cell neoplasm, high number of PB CTPC being strongly associated with a malignant disease behavior and poorer outcome of both MGUS and MM.

Sanoja-Flores et al (2018) reported that CTPC were detected in PB in 100% of MM and in 59% of MGUS patients.^[19]^ Earlier studies showed that detection of CTPC in PB in MGUS is about 19% to 37% and 50% to 75% in MM^[41,42]^ indicating the modern improvement in NGF sensitivity. In our case, NGF detected lower percentage of 0.002% with lambda monotypic PC in PB reflecting the low burden of disease in the BM. The BM lambda restricted monotypic PC accounted for 0.25%. The low burden of PC in the BM maybe underestimated due to the fact that PCs can be patchy, and they are inherently frail cells that can be lost easily during processing. Moreover, BM aspirations are historically using the first BM pull for morphology assessment and the next pulls for the IF which leads to a diluted sample when reaching the NGF study.

NGF is a method that can be utilized for MRD. Current MRD assays are becoming routine clinical practices in patients diagnosed with different malignancies.^[42]^ There is a consistent association between MRD negativity by FCM and improved progression-free and overall survival.^[43,44]^ However, MRD assays are lacking standardization with reported variability in FCM-MRD methodology and sensitivity.^[44]^ Currently, various markers and antibody panels, distinct numbers of cells measured, and MRD positivity criteria are applied worldwide.^[43,44]^ Hence, standardization efforts as well as consensus recommendations have been proposed recently.^[45–47]^ These consensus recommendations still rely on subjective “expert-shared” knowledge and experience, and do not offer complete solution for the lack of technical standardization.^[44]^

Several publications, streaming over the past decade have emerged, demonstrating enhanced prediction of outcome using flow MRD testing for MM over conventional response assessments.^[10–13,48–50]^ In these studies, FCM been shown to be an independent predictor of progression frees (PFS) and overall survival (OS). Multiparametric flow cytometry is a high potential technique that facilitates analysis of a large number of events in heterogeneous cellular specimens, thereby providing information on a cell-by-cell basis, with an ability to acquire high number of cells rapidly. Thus, flow cytometry is well suited for rare event detection in assays such as FCM MRD.

Standard therapy for systemic treatment of scleromyxedema has not been established yet. Numerous medications and methods are used with varying therapeutic effects.^[9]^ These include: topical application and intralesional injection of hyaluronidases, systemic administration of corticosteroids, radiotherapy, psoralen, and ultraviolet A (PUVA) phototherapy, plasmapheresis combined with pulsed corticosteroid and/or immunosuppressive therapy, intravenous immunoglobulin combined with thalidomide, extracorporeal photochemotherapy,^[9,51–54]^ retinoid,^[54]^ peripheral blood autologous stem cell transplantation.^[52]^ Our patient was refractory to lenalidomide however improved clinically on immunoglobulins infusions on monthly basis without change in the MGUS level. This case shows the impact of NGF in the upfront diagnosis of MGUS in SM and because of high sensitivity can be largely utilized in the follow up after therapy.

## Author contributions

**Conceptualization:** Ruba Y. Taha, Laurent Garderet, Halima El Omri.

**Data curation:** Ruba Y. Taha, Saba Hasan, Yannick Chantran, Siveen Sivaraman.

**Formal analysis:** Ruba Y. Taha, Firyal Ibrahim.

**Funding acquisition:** Ruba Y. Taha, Halima El Omri.

**Investigation:** Zhi-bin Tian, Saba Hasan, Yannick Chantran, Siveen Sivaraman.

**Methodology:** Ruba Y. Taha, Yannick Chantran, Ahmad Al Sabbagh.

**Project administration:** Ruba Y. Taha, Saba Hasan, Firyal Ibrahim, Yannick Chantran, Hesham El Sabah, Siveen Sivaraman, Issam Al Bozom, Ahmad Al Sabbagh, Laurent Garderet, Halima El Omri.

**Resources:** Saba Hasan, Firyal Ibrahim, Yannick Chantran, Siveen Sivaraman, Issam Al Bozom.

**Software:** Saba Hasan, Firyal Ibrahim, Yannick Chantran, Siveen Sivaraman, Issam Al Bozom.

**Supervision:** Ruba Y. Taha, Saba Hasan, Firyal Ibrahim, Yannick Chantran, Hesham El Sabah, Siveen Sivaraman, Issam Al Bozom, Ahmad Al Sabbagh, Laurent Garderet, Halima El Omri.

**Validation:** Ruba Y. Taha, Saba Hasan, Firyal Ibrahim, Yannick Chantran, Hesham El Sabah, Siveen Sivaraman, Issam Al Bozom, Ahmad Al Sabbagh, Laurent Garderet, Halima El Omri.

**Visualization:** Ruba Y. Taha, Saba Hasan, Firyal Ibrahim, Yannick Chantran, Hesham El Sabah, Siveen Sivaraman, Issam Al Bozom, Ahmad Al Sabbagh, Laurent Garderet, Halima El Omri.

**Writing – original draft:** Ruba Y. Taha, Saba Hasan, Yannick Chantran, Hesham El Sabah.

**Writing – review & editing:** Ruba Y. Taha, Laurent Garderet, Halima El Omri.
